# A Quantitative Approach to Biodiversity Offset Multipliers: Managing Uncertainty in Complex Projects

**DOI:** 10.1007/s00267-026-02623-w

**Published:** 2026-09-12

**Authors:** Adam S. van der Lee, Madison E. Brook, Marten A. Koops

**Affiliations:** Fisheries and Oceans Canada, Great Lakes Laboratory for Fisheries and Aquatic Sciences, Burlington, ON Canada

**Keywords:** Biodiversity offset, No net loss (NNL), Offset multiplier, Compensation ratio, Uncertainty

## Abstract

Biodiversity offsetting is a widely used strategy to mitigate residual environmental impacts from development projects, with the objective of achieving No Net Loss (NNL). However, offsetting efforts are often undermined by uncertainty in outcomes, time delays in implementation, and inconsistent valuation methods. We present a robust, quantitative framework for calculating biodiversity offset multipliers that explicitly incorporate uncertainty across multiple offsets within a project. Building on previous work, we model offsets and impacts as stochastic values and apply Monte Carlo simulations to estimate risk-based multipliers. This framework allows for strategic allocation of compensation using a weighting parameter, enabling proponents to optimize offset investments based on the costs and reliability of offsets. Simulation results demonstrate that the proposed method consistently meets defined risk tolerance thresholds across a range of ecological scenarios. By providing a transparent and adaptable tool, this approach can enhance regulatory defensibility, support timely and credible offset approvals, and strengthen the ecological integrity of NNL outcomes.

## Introduction

Biodiversity offsets have become a strategic tool to compensate for the detrimental impacts of development activities on the natural environment (Bull et al. 2016; Maron et al. 2025). They attempt to counterbalance the residual losses from activities that impact the environment with gains from conservation actions elsewhere. Offsetting is typically the final step in the mitigation hierarchy, a sequential approach employed to avoid and minimize environmental impacts. Usually, step one stipulates that harm to the environment should be avoided. If harm is unavoidable, step two requires that it be mitigated (minimized). Some hierarchies specify that the next step is to rehabilitate impacted habitat. Once all avenues of avoidance and mitigation have been exhausted, residual impacts may be offset. In many offset policies, the objective is to achieve no net loss (NNL) of affected biodiversity values (Bull et al. 2016).

Despite their widespread adoption, with more than 100 countries having enacted offsetting policies (Marshall et al. 2023), biodiversity offsetting practices have faced substantial criticism for exchanging immediate and certain ecological losses for uncertain future gains (Maron et al. 2012). These concerns are evident in Canada, where empirical assessments have revealed frequent shortcomings in offset implementation and compliance. For example, Quigley and Harper (2006a) found that 62.5% of offsets resulted in a net loss in habitat productivity, and 82% were out of compliance with their authorizations (Quigley and Harper 2006b). In Quebec, Poulin et al. (2016) reported that inadequate application of the mitigation hierarchy led to the loss of 99% of legally disturbed wetland habitat between 2006 and 2010. Similarly, Favaro and Olszynski (2017) found that 67% of reviewed projects were authorized to impact more habitat than they were required to compensate for. A recent assessment of offsetting practices applied to species at risk of extinction in Ontario found that specified benefit criteria from offset projects were not consistently met and timelines for completion were often lacking (Lysyk et al. 2026). These Canadian examples, and similar findings in other jurisdictions (zu Ermgassen 2019), underscore the need for more rigorous, transparent, and enforceable offset frameworks, particularly those that incorporate uncertainty and risk tolerance, to ensure that offsetting contributes as intended to conservation outcomes and regulatory credibility.

Offsetting can fail to achieve NNL for a variety of reasons, including a lack of equivalency between project impacts and offsetting measures, non-compliance, time delays in implementation and functionality, and uncertainty in the effectiveness of offsetting practices (Theis et al. 2022). Equivalency represents the balancing of the expected quality or value of the impacted and offset habitats when expressed with a common metric (Quetier and Lavorel 2011). Compliance is a regulatory issue and requires strict enforcement of government policies. Compliance has been shown to vary by offset project type and jurisdiction, and importantly, offset function has been shown to improve with greater compliance (Theis et al. 2019). Time delays arise when offsetting measures are not put into place until after a development project has begun or when it takes time for the ecological benefit of the offset to be realized. This can lead to an initial loss in biodiversity and an imbalance between the losses and gains of the impact and offset. Prior to implementation, offsetting plans represent predicted gains and are therefore inherently uncertain. Offset uncertainty arises from variability in the success of restoration or habitat creation efforts, as well as limitations in baseline knowledge, measurement processes, and the predictability of ecological outcomes. The degree of uncertainty can vary by offset type (e.g., habitat restoration, habitat creation; Maron et al. 2012; Theis et al. 2019) and can be complicated by natural and anthropogenic events. The ecological outcome of an offset is difficult to quantify (Maron et al. 2012), and when evaluating NNL across infrastructure projects, Weissgerber et al. (2019) found that impacts were presented in much more detail than offsetting measures. Monitoring of offset outcomes is frequently inadequate or based on suboptimal study design, leading to insufficient baseline knowledge and potentially overly optimistic assumptions regarding their effectiveness (Moilanen et al. 2024). Additionally, variation in who conducts field assessments and how they are conducted can significantly affect the valuation of offsets. Contos et al. (2025) reported an average variation of approximately 34% in offset credits due to differences in assessor interpretation and methodology. These uncertainties present a substantial barrier to achieving a balanced and reliable offset outcome.

The effects of equivalency, uncertainty, and time delays, however, can potentially be mitigated through the application of offset multipliers, where the size of the offset is increased to provide additional compensation. Offset multipliers can increase the likelihood of achieving NNL, or even net gain, in biodiversity once the offset is in place and functional. Some researchers argue that multipliers would need to exceed 100 to ensure a high degree of certainty in achieving NNL (Moilanen et al. 2009; Bull et al. 2017), while others contend that NNL may never be achievable regardless of multiplier size (Moreno-Mateos et al. 2015). Yet, there is evidence that offset multipliers below 5 may be sufficient to compensate for residual impacts of development projects, particularly in freshwater ecosystems (Quigley and Harper 2006a; Bradford 2017; Theis et al. 2022). In evaluations of offset ratios and offset performance, it is often unclear which elements of compensation are addressed by multipliers, whether uncertainty, time delays, or lack of equivalency. However, reviews of offsetting practices consistently demonstrate that “more is better” (e.g., Theis et al. 2019; zu Ermgassen 2019).

Although the use of offset multipliers to specifically account for uncertainty has long been advocated for (Moilanen et al. 2009), few examples exist of simple and robust methods for their estimation. This leaves managers without a defensible approach to apply and enforce their use. Canada’s offset policy for aquatic habitat (DFO 2025) includes a guiding principle that time lags and uncertainty must be accounted for in offset measures; however, it provides no guidance or methodology on how this should be accomplished. As a result, offset multipliers are often not required, at least partially due to the lack of a fair and robust quantitative method.

To address this gap, we present an updated method, based on Bradford (2017), to estimate offset uncertainty multipliers for complex impact-offset schemes using minimal data inputs. We test the method’s performance through simulation-based scenarios that reflect a range of ecological and implementation conditions. Our goal is to provide managers and policymakers with a simple, transparent, and defensible tool to improve the reliability of biodiversity offsetting and support the achievement of NNL.

## Uncertainty Offset Multipliers

To achieve NNL, there must be equivalency between the impacts (i.e., the residual effects from a development project) and the offset measures. At face value, offset gains are typically less than the impact losses, and equivalency is therefore achieved with a multiplier:

1 $${d}_{i}={d}_{o}m,$$

where *d*_*i*_ is the estimate of the impact value, *d*_*o*_ is the estimate of the offset value, and *m* is the multiplier that ensures equivalency. For this equation to be meaningful, both impact and offset sites must be evaluated using a common ecological currency, a standardized metric that allows for direct comparison of gains and losses (Bradford et al. 2016; Koops et al. 2022). The choice of metric is critical, as it determines both the components of biodiversity that require compensation and the scale of offset actions needed to balance losses and gains (Moilanen and Kotiaho 2018). Metric selection depends on numerous factors such as the type and location of impact, desired outcomes of offsetting, and legislative requirements (Carreras et al. 2018). Many examples of ecosystem currencies exist (Marshall et al. 2023), which can be broadly grouped into three categories: biodiversity, landscape, and ecosystem services (Borges-Matos et al. 2023). Without a common currency, equivalency assessments can be inconsistent or misleading, undermining the credibility of offsetting outcomes and regulatory decisions.

Typically, the losses and gains from the impact and offset are uncertain quantities. Uncertainty in the impact can be related to an incomplete understanding of how the habitat is affected, unknown species-specific or community responses, and variation due to measurement error during field assessments. Offset uncertainty relates to the effectiveness and efficacy of the restoration or habitat creation methods, as well as factors such as measurement error and uncertain productivity baselines.

Bradford (2017) presented a method to incorporate uncertainty when evaluating equivalency and calculating offset multipliers. Rather than fixed values, both *d*_*i*_ and *d*_*o*_ are represented as distributions with specified means and variances. Bradford (2017) used normal distributions to characterize uncertainty; however, here we apply lognormal distributions, ensuring continuous and positive quantities and allowing for greater uncertainty than the normal distribution. Additionally, lognormal distributions become increasingly right-skewed as uncertainty increases, which we believe better reflects the potential outcomes of offsetting.

The estimates of the impact and offset are now represented by distributions (denoted with capital letters), such as: ${D}_{i} \sim {lognormal}({\mu }_{i},{\sigma }_{i})$ and ${D}_{o} \sim {lognormal}({\mu }_{o},{\sigma }_{o})$. These are incorporated into Eq. 1, with the multiplier now also a distribution, *M*, calculated as:

2 $$M=\frac{{D}_{i}}{{D}_{o}}$$

This yields a distribution of potential multipliers that ensures equivalence and accounts for uncertainties, which can be estimated with Monte Carlo simulation. The applied multiplier, *m*, can be extracted from the *M* distribution based on management risk tolerance. A risk tolerance threshold, *r*, is identified, and the 1-*r* quantile of the distribution is selected ($m={M}_{1-r}$). Risk tolerances of 20% (Cochrane et al. 2015; Bradford 2017) and 5% (Moilanen et al. 2009) have been proposed. At a risk tolerance of 20%, the 80th percentile is taken from the *M* distribution and allows for a 1-in-5 chance that offsetting results in a net loss. Lower risk tolerances increase the likelihood of achieving NNL or net gain, but require larger multipliers.

Large development projects are typically complex, involving multiple impacts and offsets. For example, Weissgerber et al. (2019) indicated that, on average, there were 3.8 offset sites per project across 24 reviewed development projects from France. During project planning, it can be advantageous to model offsetting in a disaggregated manner by explicitly itemizing biodiversity elements on both the impact and offset side (Maseyk et al. 2016). Equation 2 is easily extended to accommodate multiple impacts and offsets:

3 $$M=\frac{{\sum }_{j=1}^{{n}_{i}}{D}_{i,j}}{{\sum }_{j=1}^{{n}_{o}}{D}_{o,j}}$$

where *n*_*i*_ and *n*_*o*_ are the number of impacts and offsets, respectively. This framework assumes a constant multiplier applies across all offsets. However, applying additional compensation to specific offsets may be cost-prohibitive, impractical, infeasible, or undesirable (e.g., fish stocking (Loughlin and Clark 2014)). Therefore, methods to identify offset-specific multipliers while accounting for uncertainty across all offsets are necessary.

Equation 3 can be adapted by introducing an offset weight parameter, *w*_*j*_, ranging from 0 to 1:

4 $$M=1+\frac{{\sum }_{j=1}^{{n}_{i}}{D}_{i,j}-{\sum }_{j=1}^{{n}_{o}}{D}_{o,j}}{{\sum }_{j=1}^{{n}_{o}}{w}_{j}{D}_{o,j}}$$

The weights represent the ability or willingness to apply multipliers to a given offset, where 1 indicates no limitations to increasing the offset, and 0 indicates no scope for increasing its size. Intermediate values (between 0 and 1) do not have a direct standalone interpretation, as their influence on the resulting multiplier depends on the number of offsets and the relative magnitude and uncertainty of each project. In practice, *w*_*j*_ can be adjusted iteratively to ensure that the resulting multiplier remains feasible within project-specific constraints, such as cost, logistics, and implementation capacity.

Equation 4 defines the distribution of potential multipliers. The offset-specific multipliers, *m*_*j*_, are determined by taking the 1-*r* quantile from *M* and applying:

5 $${m}_{j}=\left({M}_{1-r}-1\right){w}_{j}+1$$

The multipliers in Eq. 5, *m*_*j*_, explicitly account for both the need to achieve equivalency between impacts and offsets and the uncertainty associated with their estimated values. Identifying offset-specific multipliers in this manner ensures that required compensation reflects not only differences in ecological value but also the variability and risk inherent in both impact and offset outcomes, up to the specified level of risk tolerance.

## Simulations and Results

We used simulation analysis to explore the efficacy of this approach across multiple offsets with varying levels of uncertainty, offset weights, and covariances. As in Bradford’s (2017) approach, covariance represents the level of dependence in the impact and offset uncertainties. No covariance implies the uncertainties are independent, whereas high covariance indicates that the stochastic draws used to generate uncertainty distributions are strongly correlated. Covariance may occur among individual impacts or among offsets, as well as between impacts and offsets. This can arise, for example, when impacts and offsets are located in the same area and are influenced by shared environmental drivers or stochastic events, such as climatic fluctuations. In practice, however, high covariance is likely to be uncommon.

Monte Carlo simulations were conducted with 100,000 replicates of random draws of impacts and offsets based on the specified distributions, uncertainties, and covariances. Expected impacts and offsets were represented as arithmetic means (e.g., $\bar{{D}_{i}}$, $\bar{{D}_{o}}$) with equivalency between impacts and offsets maintained such that ${\sum }_{j=1}^{{n}_{i}}\bar{{D}_{i,j}}={\sum }_{j=1}^{{n}_{o}}\bar{{D}_{o,j}}$. Thus, multipliers in our simulations account for uncertainty alone.

Uncertainty was represented using the coefficient of variation (CV = σ/μ) and modeled with lognormal distributions ($D \sim {lognormal}(\mu ,\sigma )$), where $\mu =\log \left(\bar{D}\right)-\frac{{\sigma }^{2}}{2}$ and $\sigma =\sqrt{\log ({{CV}}^{2}+1)}$. The CV is a dimensionless measure of variability, ensuring that simulation results (i.e., offset multipliers) are comparable across analyses and not dependent on the choice of equivalency metric. Simulations used CV values ranging from 0.1 to 1 to represent low to high levels of uncertainty. However, actual uncertainty will depend on project type, data availability, and practitioner experience; therefore, what constitutes low or high uncertainty may vary and should be informed or validated through independent analysis.

Correlated uncertainty was generated by drawing values from a multivariate normal distribution with a specified correlation structure, transforming these to uniform probabilities, and then mapping them to lognormal distributions parameterized by the specified mean and CV for each impact and offset.

All analyses were conducted in R 4.4.1 (R Core Team 2024).

### Is NNL Achieved at the Set Risk Tolerance?

We first examined whether this approach achieves NNL at designated risk tolerance levels (r = 0.2, 0.1, 0.05). Monte Carlo simulations were run across different offset uncertainties (CV = 0.25, 0.5, or 1), different numbers of offsets (*n*_*o*_ = 1, 2, or 3), and varying levels of covariance among impact and offsets (ρ = 0, 0.5, 0.95). Uncertainty was constant across all offsets, and there were no limits to compensation (*w* = 1). Simulations included one impact with CV_i_ = 0.25.

The probability of no net loss (P[NNL]) was determined as the proportion of replicates (N) where the total gains from offsets equaled or exceeded the impacts:

6 $$P\left[{NNL}\right]=\frac{\sum \left({\sum }_{j=1}^{{n}_{i}}{D}_{i,j}\le {\sum }_{j=1}^{{n}_{o}}{m}_{j}{D}_{o,j}\right)}{N}$$

P[NNL] was calculated with and without uncertainty multipliers applied to the offsets.

**Fig. 1:**
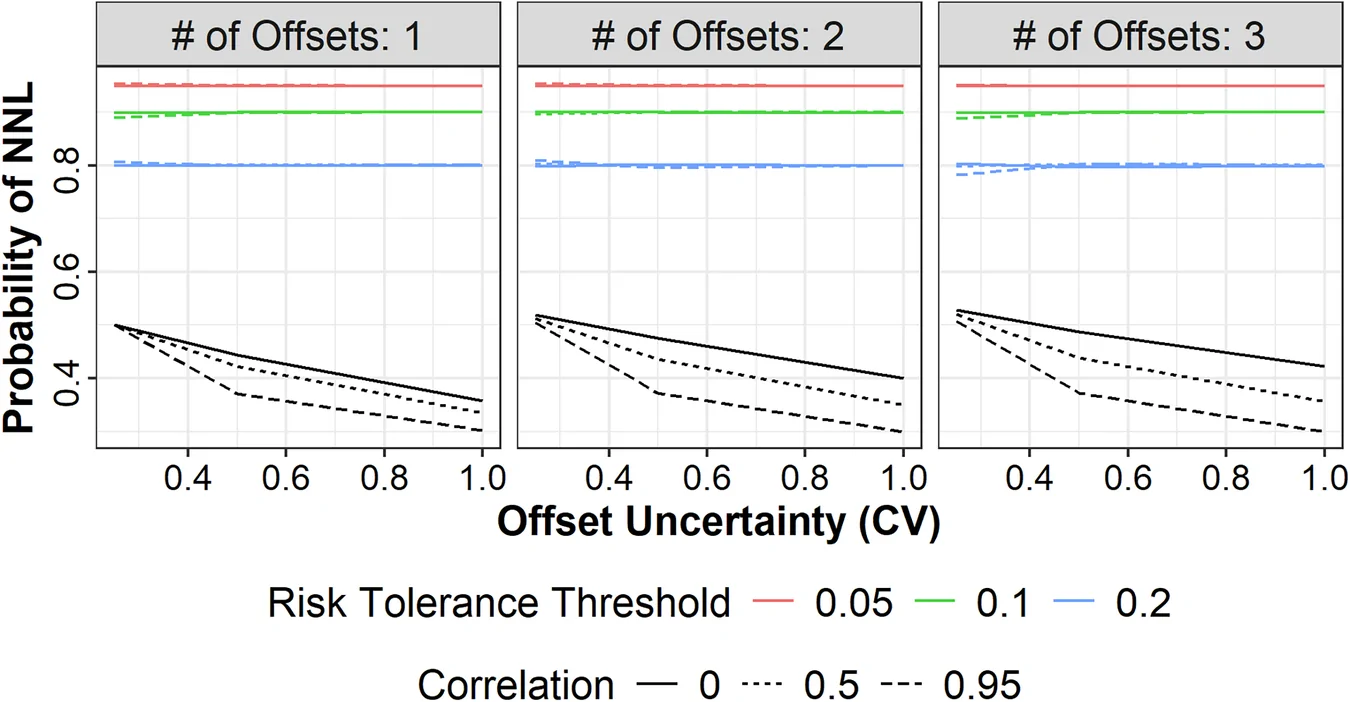
Simulation results illustrating the effectiveness of achieving no net loss (NNL) under varying conditions of offset uncertainty. Black lines represent scenarios without offset multipliers, while colored lines show outcomes with multipliers applied at different risk tolerance levels. Line type indicates covariance between impact and offsets, and panels represent the number of offsets (1 to 3). Impact uncertainty is held constant at CV = 0.25

With uncertainty multipliers applied, NNL was achieved at the designated risk tolerance across scenarios, regardless of the number of offsets, offset uncertainty, or covariance (Fig. 1). In contrast, NNL was achieved only ~50% of the time when uncertainty multipliers were not applied, and this decreased with increased offset uncertainty and covariance. The minimum P[NNL] was ~30% when offset uncertainty was high (CV_o_ = 1), and covariance was high (ρ = 0.95). Across scenarios, the uncertainty multipliers ranged from 1.05 (*r* = 0.2, CV_o_ = 0.25, ρ = 0.95) to 6.98 (*r* = 0.05, CV_o_ = 1, ρ = 0).

### How Do Offset Weights (w_j_) Affect Multipliers?

Next, we examined how changing offset weightings (*w*_*j*_) affects P[NNL] and multipliers under different uncertainty and risk tolerance (*r* = 0.2, 0.1, 0.05) scenarios. These simulations include a single impact with CV_i_ = 0.25 and two offsets with no covariance (ρ = 0). We tested two offset uncertainty scenarios: (1) equal uncertainty (CV_o1_ = CV_o2_ = 0.5) and (2) unequal uncertainty (CV_o1_ = 1.0 and CV_o2_ = 0.1). We tested three offset weight scenarios: 1. the multiplier applied to both offsets (*w*_*o1*_ = *w*_*o2*_ = 1), 2. the multiplier applied only to offset 1 (*w*_*o1*_ = 1 and *w*_*o2*_ = 0), and 3. The offset multiplier applied only to offset 2 (*w*_*o1*_ = 0 and *w*_*o2*_ = 1).

**Fig. 2:**
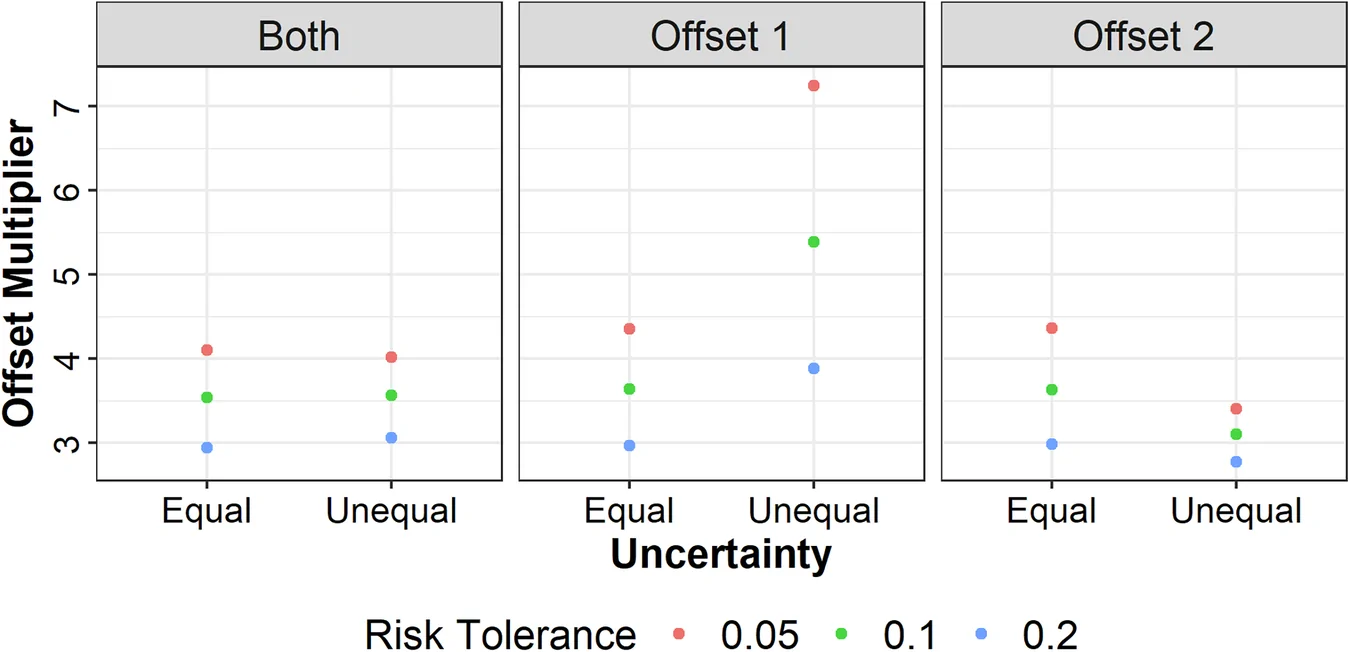
Simulation results showing how multiple offsets and offset weight affect the magnitude of multipliers. Each scenario includes a single impact (mean = 1, CV_i_ = 0.25) and two offsets (mean = 0.5). Offset uncertainty is either equal (CV_o1_ = CV_o2_ = 0.5) or unequal (CV_o1_ = 1.0, CV_o2_ = 0.1). The y-axis represents the total offset multiplier summed across both offsets (*m*_*total*_ = *m*_*1*_ + *m*_*2*_). Panels indicate how multipliers are applied: equally to both offsets (Both), only to offset 1 (Offset 1), or only to offset 2 (Offset 2). Point color denotes the level of risk tolerance

Across all scenarios, P[NNL] was achieved at the designated risk tolerance. However, the magnitude of the uncertainty multiplier was influenced by the uncertainty and weighting scenarios (Fig. 2). We examined the summation of uncertainty multipliers across the two offsets (*m*_*total*_ = *m*_*1*_ + *m*_*2*_) to represent the total compensation required regardless of the offset to which the multiplier is applied.

When the multiplier was applied to both offsets, the total compensation was similar across equal and unequal uncertainty scenarios, with minor differences due to variance. When the multiplier was applied to only one offset, large differences emerged (Fig. 2). For equal uncertainty, total compensation increased slightly when one offset received the multiplier (e.g., summed multiplier, *m*_*total*_, at *r* = 0.05 was 4.14 when applied to both offsets and 4.35 when applied to one). For unequal uncertainty, applying the multiplier to the less uncertain offset (offset 2) required less compensation (*m*_*total*_ = 3.39), while applying it to the more uncertain offset (offset 1) required more than twice the compensation (*m*_*total*_ = 7.22).

### How Does Covariance Among Impacts and Offsets Affect Multipliers?

Covariance among impacts and offsets may occur when they are subject to the same stochastic events, e.g., if the offsets are located near the impacted area, particularly when offsets are in-kind. We used simulations to explore how these covariances affect offset multipliers. Simulations included three impacts and three offsets, each with uncertainties quantified as CVs (CV_i_ = 0.1, 0.5, or 1 and CV_o_ = 0.1, 0.5, or 1), with constant CVs across impacts and across offsets, but with CVs potentially differing between impacts and offsets. All offsets had full scope for increased compensation (*w*_*j*_ = 1). Risk tolerances of 20%, 10%, and 5% were considered, and covariance (*ρ*) among impacts and offsets was varied between 0 and 1.0.

**Fig. 3:**
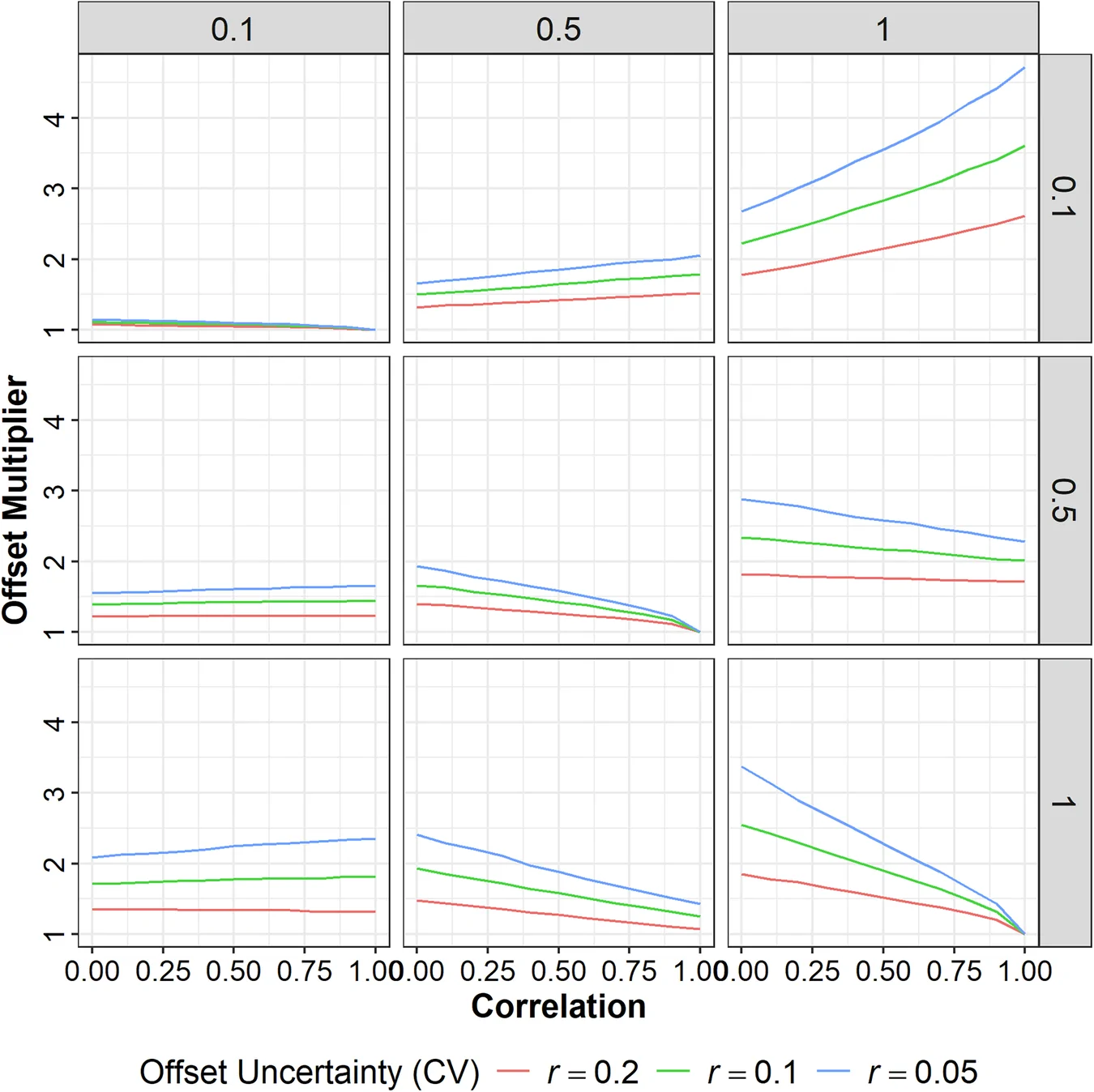
Simulation results showing the influence of covariance among impacts and offsets on the offset multiplier across risk tolerance (*r*) levels. Line types represent levels of risk tolerance, rows represent impact uncertainty (CV_i_), and columns represent offset uncertainty (CV_o_)

The effect of covariance on the offset multiplier varied depending on the relative uncertainty between the impacts and offsets (Fig. 3). When the uncertainty was equal, the multiplier decreased toward 1 as the covariance increased. High covariance reduced the effect of uncertainty and the need for additional compensation because the stochastic deviations in impacts and offsets were closely aligned. At *ρ* = 1, and with equal means and CVs, the random deviations are identical and therefore cancel out, resulting in a multiplier of *m* = 1. Although this scenario is biologically unrealistic, as impacts and offsets are unlikely to experience perfectly correlated ecological responses or identical sources of uncertainty, it highlights this important property of the modeling approach.

However, when offset uncertainty was much greater than impact uncertainty (e.g., CV_o_ = 1.0 and CV_i_ = 0.1), the multiplier required to achieve equivalency increased substantially. In these cases, multipliers were 1.6–1.74 times larger when the covariance was 1 compared to 0. Under high covariance and unequal uncertainty (CV_o_ > CV_i_), the stochastic deviations in offsets are consistently larger than those in impacts, resulting in a systematic imbalance that leads to larger multipliers. Otherwise, covariance had minimal impact on multipliers.

### How Does Uncertainty Drive Multiplier Magnitude?

Uncertainty in impacts and offsets can be difficult to quantify. We therefore explored a broad range of uncertainty combinations to inform expected multipliers. Simulations were run with CV_i_ and CV_o_ ranging from 0 to 1, and risk tolerances of 20%, 10%, and 5% (*r* = 0.2, 0.1, 0.05). Equivalency in expected impacts and offsets was maintained (${\sum }_{j=1}^{{n}_{i}}\bar{{D}_{i,j}}={\sum }_{j=1}^{{n}_{o}}\bar{{D}_{o,j}}$). Impacts and offsets had no covariance (ρ = 0), and all offsets had scope for additional compensation (*w*_*j*_ = 1).

**Fig. 4:**
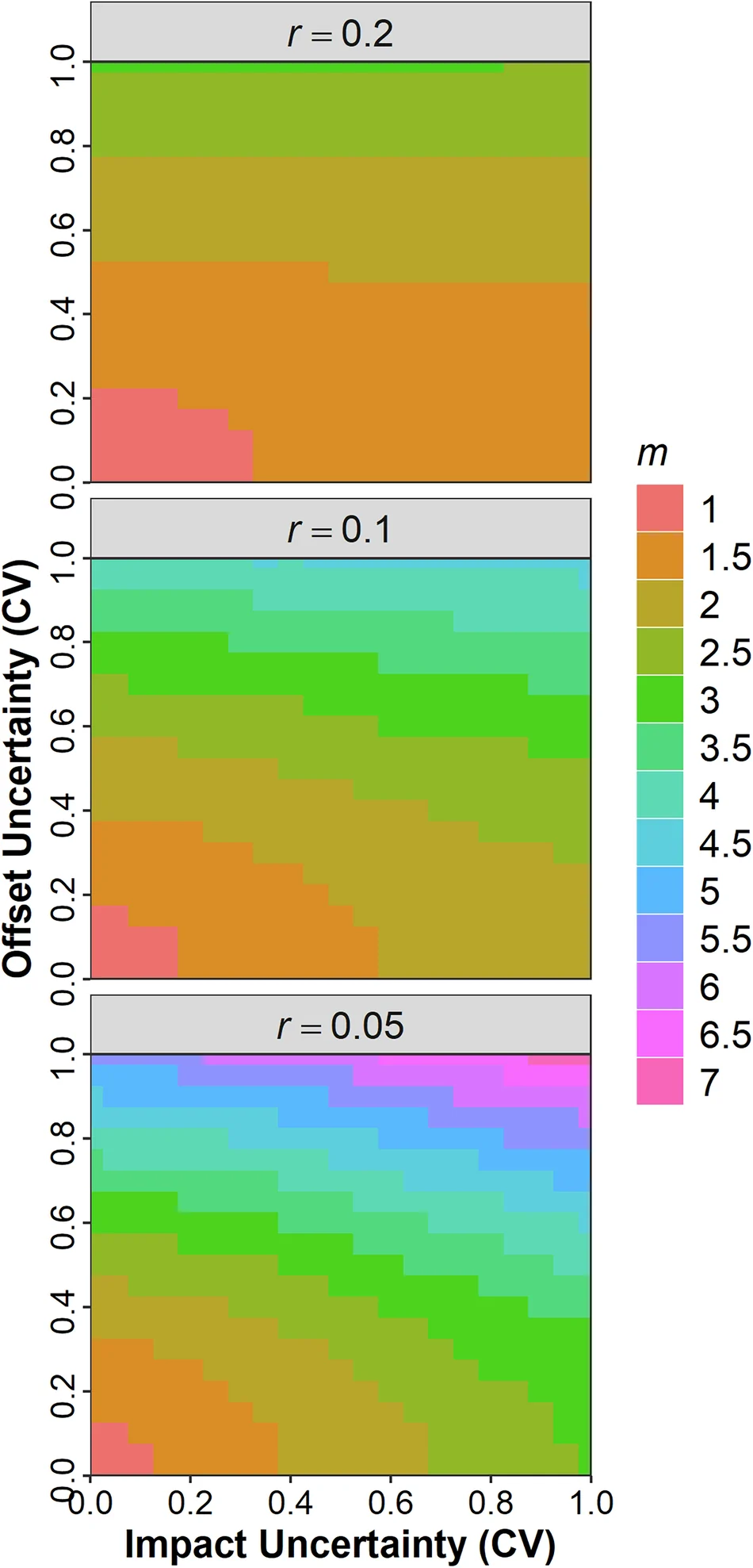
Estimated uncertainty offset multipliers across combinations of impact (x-axis) and offset (y-axis) uncertainty, expressed as coefficients of variation (CV), under three levels of risk tolerance (panel rows). Simulations assume equivalency in expected impacts and offsets, no covariance between impacts and offsets (ρ = 0), and full scope for compensation across all offsets (*w*_*j*_ = 1). These results provide a reference for selecting appropriate multipliers based on project-specific uncertainties and risk tolerance

Offset multipliers accounting for uncertainty alone ranged as high as ~3 for high risk tolerance (*r* = 0.2) to ~7 at low risk tolerance (*r* = 0.05) (Fig. 4). Greater offset uncertainty resulted in larger multipliers. At low risk tolerance, impact uncertainty had little influence on the multiplier, especially when offset uncertainty was high. Figure 4 can serve as a rule-of-thumb guide for selecting offset multipliers to account for uncertainty at a given level of risk tolerance.

## Discussion

This study introduces a novel framework for calculating uncertainty offset multipliers that explicitly incorporates risk tolerance, ecological variability, and offset-specific weightings. Unlike previous approaches that rely on fixed ratios or simple equivalency assumptions, our method accounts for uncertainty by using probabilistic modeling to align offset requirements with defined levels of acceptable risk across multiple offsets. This represents a conceptual advance in biodiversity offsetting in complex projects, offering a transparent and adaptable mechanism for achieving NNL and potentially net gain in biodiversity under uncertainty. This framework provides a practical way to incorporate uncertainty and risk into offsetting decisions. For example, in Canada, the current offset policy (e.g., DFO 2025) emphasizes the need to account for uncertainty but does not prescribe a quantitative method. As well, our approach is sufficiently flexible to be integrated into existing offset calculators and regulatory frameworks across jurisdictions.

The simulation results validate the robustness of this framework across a range of plausible scenarios. By modeling offset outcomes using lognormal distributions and varying degrees of covariance, we demonstrate that multipliers can consistently meet risk-based performance thresholds. These findings suggest the method is not only theoretically sound but also practically applicable in diverse environmental and regulatory contexts.

Numerous frameworks have been developed to balance ecological losses and gains in biodiversity offsetting through multipliers, often incorporating a broad range of considerations relevant to offset planning (Moilanen and Kotiaho 2018). In some cases, uncertainty is acknowledged but not explicitly quantified, with multipliers recommended but without clear guidance on how they should be estimated (DFO 2025, Moilanen and Lehtinen 2025). Where uncertainty is incorporated explicitly, it is often parameterized using expert judgment. For example, uncertainty may be represented through probabilities of success over a specified time horizon, reducing the expected value of offsets (Miller et al. 2015; Maseyk et al. 2016), or through approaches such as info-gap theory that bound potential outcomes around an expected value (Moilanen et al. 2009). While these approaches provide structured ways to account for uncertainty, they can lack transparent and consistent parameterization, with outcomes dependent on subjective inputs that may vary among practitioners. The method presented here provides a complementary approach that could be readily incorporated into existing frameworks, offering several practical advantages. These include the ability to disaggregate impacts and offsets across multiple biodiversity components, the use of explicitly defined probability distributions to represent uncertainty, and the application of clearly defined risk tolerance thresholds to guide decision-making. Importantly, the framework also incorporates uncertainty in both impacts and offsets, allowing for more robust outcomes in cases where impacts may be underestimated.

While we have presented a simple parameterization of the model using the expected mean and CV of lognormal distributions for each impact and offset, identifying these parameters in practice is non-trivial. Accurately valuing impacted areas will require pre-impact sampling (Moilanen et al. 2024) with sufficient replication to quantify uncertainty. Similarly, estimating the expected value and uncertainty of offsets prior to implementation will depend on detailed knowledge of outcomes from comparable projects, as well as practitioner expertise. Following implementation, monitoring of offset performance and rigorous accounting of gains and losses will be required to ensure, at a minimum, the achievement of NNL, preferably using a robust study design (e.g., Before-After-Control-Impact or BACI design) (Moilanen et al. 2024). Although correlation among impacts and offsets may occur, correlations are unlikely to be strong and will be difficult to reliably quantify in most cases. Moreover, as the number of impacts and offsets increases, so too does the complexity of the covariance structure, resulting in a rapidly growing number of correlation terms that must be specified. Thus, while covariance was an important modeling feature, it is unlikely to be relevant in practical applications. Therefore, unless supported by strong empirical evidence, we recommend assuming independence (i.e., ρ = 0) when applying this analysis. This precautionary approach avoids introducing poorly supported covariance assumptions that could influence offset requirements without sufficient empirical justification.

In the future, it may be possible to broadly categorize project types into qualitative uncertainty classes based on expected ranges of CV (e.g., low: CV < 0.25; moderate: 0.25 ≤ CV < 0.5; high: CV ≥ 0.5). The empirical basis for such classifications, however, is currently limited, and further synthesis of monitoring data across projects will be required before these generalizations can be applied confidently.

We expect the outcomes of impacts and offsets to be well represented by a lognormal distribution; however, the choice to parameterize these distributions using a mean and CV was largely driven by convenience for incorporation into simulation analyses. Our proposed framework is flexible and can accommodate any biologically reasonable distribution and parameterization, including cases where distributional choices vary among individual impacts or offset projects within a scheme. For data-limited situations, an attractive alternative is the PERT distribution (Malcolm et al. 1959), which, similar to the triangular distribution (Evans et al. 2000), offers a flexible shape and is parameterized using minimum, most likely (mode), and maximum values. These parameters may be more readily specified in practice than the mean and variance, particularly when empirical data are limited.

We provide a simplified hypothetical example to demonstrate application of the framework. Consider a development project that will impact 5 ha of high-quality riparian habitat. Habitat value is normalized to *d*_*i*_ = 1.0, the impacted area is fixed at 5 ha, and confidence in the estimate is high, such that *CV*_*i*_ = 0.1. The proposed offset includes two projects: (1) riparian restoration in a nearby location, and (2) construction of off-channel habitat (e.g., a pond). These offset options differ in both ecological productivity and construction cost (Theis et al. 2022). For the riparian restoration project, there is insufficient information to parameterize a lognormal distribution; therefore, a PERT distribution is used. The most likely value is set at 0.2 (i.e., 20% as productive as the impacted habitat; *d*_*o1*_ = 0.2), with a minimum value of 0 (no productivity; *min*_*o1*_ = 0.0) and a maximum value of 1.0 (equivalent to the impacted habitat; *max*_*o1*_ = 1.0). The construction cost is estimated at $17/m² (Theis et al. 2022). The habitat creation project has the potential to achieve productivity comparable to the impacted habitat (*d*_*o2*_ = 1.0), but with much higher uncertainty (*CV*_*o2*_ = 0.5). Regulators require a relatively high level of confidence in achieving no net loss, with a risk tolerance of *r* = 0.1. The framework can be used to estimate the offset area required to achieve this level of confidence while also evaluating cost implications. If equal weighting is applied to both offset projects (*w*_*1*_ = *w*_*2*_ = 1), each project would require 6.89 ha (13.78 ha in total) to offset the 5 ha of impacted habitat, at an estimated cost of $7.03 M. If only riparian restoration is used (offset 1), 55.7 ha would be required, at a cost of $9.47 M. Alternatively, if only habitat creation is used (offset 2), 10.31 ha would be required, at a cost of $8.76 M. By adjusting the weighting parameters (*w*_*j*_) to reflect both ecological and practical constraints, the framework can identify cost-effective offset strategies. In this example, the cost-minimizing solution is obtained with *w*_*1*_ = 1 and *w*_*2*_ = 0.28, resulting in multipliers of 14.4 and 4.9, respectively (19.3 ha total), at a total cost of $6.58 M. This illustrates how the framework can identify cost-efficient offset strategies by balancing differences in productivity, uncertainty, and cost, and demonstrates that a mixed-offset approach can outperform strategies that rely on a single offset type. To allow users to further explore multiplier calculations using our framework, we have developed an interactive Shiny application (see “Data Availability” section).

An assumption inherent in this framework is that both the proponent and the regulator are acting in good faith with the common objective of balancing the losses and gains from impacts and offsets (i.e., NNL). Regulators are not punishing proponents for others’ poor performance; nor are proponents attempting to minimize their obligations by underestimating impacts and overestimating offsets. The application of uncertainty multipliers is in the best interest of both well-intentioned proponents and regulators, as uncertainty is inherent in the complexity of natural ecosystems.

For regulators, the proposed framework offers a more transparent, defensible, and consistent approach to evaluating biodiversity offsets. By explicitly incorporating uncertainty and risk tolerance into multiplier calculations, it moves offsetting away from subjective or inconsistent practices and toward a quantitative standard that can be applied across projects. This can enhance the credibility of offset approvals and strengthen the regulatory basis for enforcing NNL outcomes.

From the proponent’s perspective, applying uncertainty offset multipliers offers several strategic advantages. First, it provides greater cost certainty by quantifying the level of compensation required to meet regulatory expectations under defined risk tolerances. This allows proponents to incorporate offset requirements into project planning with a clearer understanding of scope and budget. Second, the use of multipliers increases the likelihood of satisfying authorization conditions, reducing the risk of non-compliance and associated delays or penalties. Third, a robust and transparent method for incorporating offset multipliers may expedite the regulatory approval process. When proponents demonstrate a scientifically defensible approach for managing uncertainty, regulators can more confidently and efficiently assess project proposals. This can lead to faster decision-making and reduced administrative burden. Our approach provides a clear and predictable application of the polluter-pays principle, ensuring that proponents account for uncertainty in outcomes from the outset while reducing the risk of non-compliance. While this may result in outcomes that exceed NNL, any resulting net gain reflects a precautionary buffer rather than a direct benefit to the proponent.

The proposed method accounts for uncertainty across multiple impacts and offsets, offering a flexible and strategic framework for biodiversity offsetting. By incorporating a weighting parameter, proponents can optimize offset allocation across sites with varying costs and ecological uncertainties (e.g., Theis et al. 2022). This enables targeted investment in offsets that are both cost-effective and ecologically reliable, enhancing the likelihood of regulatory compliance while managing financial burden. Outside of the boundary cases where *w*_*j*_ = 1 (no constraint on compensation) or *w*_*j*_ = 0 (no capacity for additional compensation), the value of *w*_*j*_ is generally not known a priori. Instead, the analysis can be conducted iteratively, adjusting *w*_*j*_ until the resulting multiplier satisfies project-specific constraints and is acceptable to the regulator. Importantly, this approach supports and emphasizes bet-hedging, whereby offset efforts are distributed across multiple sites to reduce the probability of complete failure (van Katwijk et al. 2009; Moilanen et al. 2009), thereby improving the resilience of offset outcomes. The ability to tailor offset strategies using weighted multipliers provides proponents with a powerful decision-support tool, enabling them to navigate ecological and economic trade-offs more effectively, especially under increasing scrutiny and urgency in project approvals.

While our method offers a structured approach to managing uncertainty in biodiversity offsetting, it does not address all risks inherent in offsetting. Notably, it cannot compensate for the complete failure of an offset (Moilanen et al. 2009, Maron et al. 2012), as no multiplier can rectify total ecological loss. Moreover, the framework assumes good faith actions by proponents and regulators, and does not account for scenarios involving negligence or deliberate non-compliance. Of particular concern are poorly or underestimated impacts (Quigley and Harper 2006a, Josefsson et al. 2021), which can disproportionately affect sensitive species or habitats (Dey et al. 2024; Brook et al. in review). Finally, the framework does not guarantee the permanence of offsets, which may deteriorate over time due to a lack of maintenance or changing environmental conditions, ultimately reducing their ecological functionality (Souza et al. 2023). Such risks must be managed through ongoing monitoring, site maintenance, and adaptive management.

In addition to ecological uncertainty, time delays in the implementation of offsetting measures must be carefully considered, as they can affect offset effectiveness (Moilanen and Kotiaho 2018). These delays may be mitigated with additional compensation through the use of a time-delay multiplier (Laitila et al. 2014; Bradford 2017). This multiplier is derived by comparing the schedule of ecological impacts to the expected schedule of offset implementation over a defined time horizon, the period during which gains and losses are evaluated. Minns (2006) recommended using a time horizon equal to twice the duration of the project or twice the time required to fully implement offset measures. Additionally, time discounting should be routinely applied (Moilanen and Kotiaho 2018), where future ecological gains are discounted to the time of impact. This reflects the principle that delayed benefits are less certain and less valuable. Neglecting time discounting can disadvantage conservation efforts, especially when offset benefits are projected far into the future, and their realization remains uncertain (Moilanen et al. 2009). Uncertainty and time-delay multipliers are independent, meaning the total additional compensation required is the product of both multipliers.

Ideally, biodiversity offsets should be in place and achieving the desired ecological function prior to project impacts to ensure a high degree of certainty in achieving NNL (Bekessy et al. 2010). This allows for the accrual of biodiversity gains to be demonstrated before it is used to compensate for losses, reducing reliance on assumptions and improving environmental accountability. As well, offsets are inherently difficult to quantify (Maron et al. 2025), and assessments benefit from multiple comparisons across ecological states, rather than relying on a single metric (Weissgerber et al. 2019). In many cases, pre-impact implementation is not pursued due to regulatory, financial, or logistical constraints. In such situations, our proposed method provides a practical and scientifically defensible alternative, enabling offset planning that remains transparent, adaptable, and aligned with NNL objectives, even when implementation occurs after impacts.

## Data Availability

The Shiny application and R code used to perform the simulations are available on Github: https://github.com/KoopsLab/Offset_Uncertainty.
